# Real-world accuracy of computed tomography in patients admitted with small bowel obstruction: a multicentre prospective cohort study

**DOI:** 10.1007/s00423-023-03084-z

**Published:** 2023-08-29

**Authors:** L. B. J. Nielsen, M. P. Ærenlund, M. Alouda, M. Azzam, T. Bjerke, J. Burcharth, C. B. Dibbern, T. K. Jensen, J. Q. Jordhøj, I. Lolle, T. Malik, L. Ngo-Stuyt, E. Ø. Nielsen, M. Olausson, A. P. Skovsen, M. A. Tolver, H. G. Smith

**Affiliations:** 1 Abdominalcenter K, Bispebjerg and Frederiksberg Hospital, University of Copenhagen, Bispebjerg Bakke 23, 2400 Copenhagen, Denmark; 2 Department of Gastrointestinal and Hepatic Diseases, Surgical Division, Copenhagen University Hospital - Herlev and Gentofte Hospital, Copenhagen, Denmark; 3 Department of Surgery, Slagelse Hospital, Slagelse, Denmark; 4 Department of Surgery, Nordsjællands Hospital, University of Copenhagen, Copenhagen, Denmark; 5 Department of Surgery, Hvidovre Hospital, University of Copenhagen, Copenhagen, Denmark; 6 Department of Surgery, Zealand University Hospital, Koge, Denmark

**Keywords:** Small bowel obstruction, Emergency surgery, General surgery, Computed tomography

## Abstract

**Purpose:**

Small bowel obstruction (SBO) is a common surgical emergency. Previous studies have shown the value computed tomography (CT) scanning in both confirming this diagnosis and identifying indications for urgent surgical intervention, such as strangulated bowel or closed loop obstructions. However, most of the literature is based on retrospective expert review of previous imaging and little data regarding the real-time accuracy of CT reporting is available. Here, we investigated the real-world accuracy of CT reporting in patients admitted with SBO.

**Methods:**

This was a multicentre prospective study including consecutive patients admitted with SBO. The primary outcomes were the sensitivity and specificity of CT scanning for bowel obstruction with ischaemia and closed loop obstruction. Data were retrieved from the original CT reports written by on-call radiologists and compared with operative findings.

**Results:**

One hundred seventy-six patients were included, all of whom underwent CT scanning with intravenous contrast followed by operative management of SBO. Bowel obstruction with ischaemia was noted in 20 patients, with a sensitivity and specificity of CT scanning of 40.0% and 85.5%, respectively. Closed loop obstructions were noted in 26 patients, with a sensitivity and specificity of CT scanning of 23.1% and 98.0%, respectively.

**Conclusions:**

The real-world accuracy of CT scanning appears to be lower than previously reported in the literature. Strategies to address this could include the development of standardised reporting schemas and to increase the surgeon’s own familiarity with relevant CT features in patients admitted with SBO.

## Introduction

Small bowel obstruction is a common general surgical emergency [1, 2]. The management of patients with small bowel obstruction (SBO) is complicated by the heterogeneity of this patient group not only in terms of the underlying aetiology of obstruction but also in terms of the severity of the clinical presentation and the patient’s comorbidities [3, 4]. There are two main treatment strategies for SBO. The first, and most commonly utilised, is a non-operative strategy, which is successful in more than 70% of patients [5, 6]. However, depending on the cause and severity of a patient’s obstruction, an operative strategy may be more appropriate. Clear indications for operative treatment of SBO include the suspicion of bowel ischaemia or closed loop obstructions, where the blood supply to the affected bowel can be compromised [7].

Stratifying patients to these different treatment strategies can be challenging, and variation in treatment patterns for SBO has been noted both within and between nations [3, 4, 8, 9]. Computed tomography (CT) scans form a key component of the diagnostic work-up of many acute surgical conditions, and much work has been done to determine their utility in identifying patients with SBO who would benefit from early surgical intervention [10]. These studies found that CT has a high specificity and sensitivity for confirming the diagnosis of SBO, determining the presence of bowel ischaemia, and identifying the underlying aetiology.

While these data would suggest that CT could be a valuable tool in facilitating the early stratification of patients to operative or non-operative treatment, a major limitation is that the majority of these studies are based on a retrospective review of historical CT scans by a small number of expert radiologists [10]. The extent to which these retrospective data can be extrapolated to the everyday clinical environment is unknown. This study aimed to prospectively investigate the real-world accuracy of CT scan reporting in patients acutely admitted with SBO.

## Methods

This was a post hoc analysis of the Danish Audit of Small Bowel Obstruction (DASBO) study cohort. DASBO was a multicentre prospective cohort study that included consecutive patients admitted with SBO at six acute hospitals in Denmark. The primary endpoints of this study have been reported [4]. The original inclusion criteria for the DASBO study were patients aged ≥ 18 years with a radiological or clinical diagnosis of SBO. Consecutive eligible patients were included over a 4-month period. The study was registered on clinicaltrials.gov (NCT04750811), study approval was provided by the Danish Data Protection Agency (P-2021-70), and consent was obtained from all participating patients. The current study includes those patients from the original DASBO cohort who had undergone CT scanning with intravenous contrast prior to operative treatment of SBO. Patients who were successfully treated non-operatively were excluded as were those who underwent CT scanning without intravenous contrast. The study is reported according to STROBE guidelines [11].

The primary endpoints for this study were the sensitivity and specificity of CT scanning for bowel obstruction with ischaemia and closed loop small bowel obstruction. Secondary endpoints included the sensitivity and specificity of CT scanning in identifying the aetiology of SBO (adhesions, hernia, or malignancy) and, in those with adhesional obstruction, the type of adhesions (band adhesion versus dense adhesions). All patients undergoing operative management were included in the analyses regarding closed loop obstruction and SBO aetiology. However, to account for the potential development of bowel ischaemia during a trial of non-operative management, only those patients undergoing acute operation (< 24 hours) of CT-based diagnosis of SBO were included in the analyses regarding bowel ischaemia.

This analysis was performed using prospectively collected data retrieved from electronic patient records and entered in a pseudonymised format into a secure REDCap database housed by The Capital Region of Denmark, which was only accessible to the study team. CT findings were retrieved from the original radiology reports and formatted as a categorical variable for the study’s primary endpoints: radiological suspicion of bowel ischaemia — yes/no; radiological suspicion of closed loop obstruction — yes/no. The on-call radiology services at each of the involved hospitals are staffed by both trainee and consultant radiologists. Trainee radiologists must have at least 18 months experience before joining the on-call rota and have a consultant radiologist available for supplementary review of images if needed. Radiology report findings were compared with operative findings retrieved from the original operation notes. Other variables retrieved included patient demographics, history of previous abdominal surgery and/or SBO, serum inflammatory markers at the time of admission, the time from CT scanning to the start of the operation, and the aetiology of SBO.

Statistical analyses included the calculation of the sensitivity, specificity, positive predictive value, and negative predictive value of CT scanning relating to the study endpoints. Descriptive statistics comparing clinicopathological demographics between patients with and without SBO with bowel ischaemia were performed using the chi-square test for categorical variables and the Kruskal Wallis test for continuous variables. All analyses were performed using GraphPad Prism version 9.0 (GraphPad Software, San Diego, CA, USA).

## Results

### Patient characteristics

**Fig. 1:**
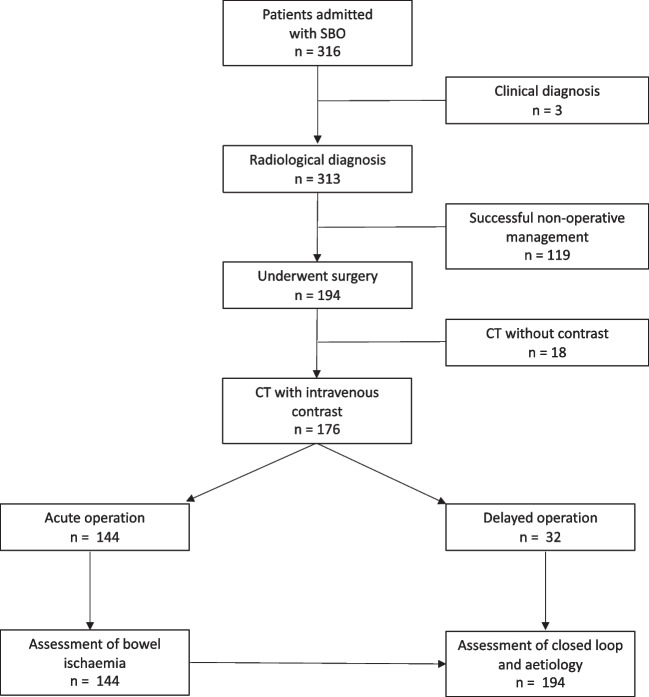
CONSORT diagram for the study cohort. SBO, small bowel obstruction; CT, computed tomography

The original DASBO study included 316 patients. Of these, 152 (48.1%) were initially managed non-operatively, with the remaining 164 patients undergoing acute operations (51.9%). The success rate of non-operative management was 78.9%. CT scanning was performed in almost all patients (313/316), of whom 176 met the inclusion criteria for the current study (Fig. 1). The majority of patients were female (93 patients, 52.8%), and the median age was 73 years (interquartile range 59-79). Adhesions were the most common cause of SBO (81 patients, 46.0%), and the majority of patients underwent an acute operation (144 patients, 81.8%). In those patients undergoing acute operations, 20 (13.9%) were found to have bowel ischaemia at the time of surgery, all of whom underwent a bowel resection. Closed loop obstructions were identified in 26 patients who underwent acute or delayed operation (14.8%). The diagnostic performance of reported CT findings in identifying bowel ischaemia, closed loop obstructions, and the aetiology of obstruction is summarised in Table 1.

**Table 1:** Diagnostic performance of computed tomography scanning in identifying bowel ischaemia and closed loop obstruction in patients admitted with small bowel obstruction

	Patients analysed	Patients with the condition	Sensitivity (%)	Specificity (%)	PPV (%)	NPV (%)
Bowel ischaemia*	144	20	40.0	85.5	30.8	89.8
Closed loop obstruction^§^	176	26	23.1	98.0	66.7	88.0
Adhesional SBO^§^	176	81	84.0	67.0	68.7	82.9
Band adhesion^#^	81	40	67.5	47.5	56.3	59.4
Hernia-related SBO^§^	176	23	95.7	98.0	88.0	99.3
Malignant SBO^§^	176	23	60.9	100	100	94.4

*SBO* small bowel obstruction, *PPV* positive predictive value, *NPV* negative predictive value

^§^Including all patients undergoing operative treatment

*Only including patients undergoing acute operation (< 24 h)

^#^Only including patients with adhesional small bowel obstruction

### Small bowel obstruction with bowel ischaemia

In patients undergoing acute operations, the median time from CT scanning to the start of the operation was 267 min (IQR 181-399). No significant difference in the time from scanning to surgery was noted between patients with and without bowel ischaemia (median 244 min (IQR 155–384) versus 268 min (192–408), *p* = 0.446). Of the 20 patients found to have ischaemic bowel at the time of surgery, the initial CT was reported to demonstrate suspicion of ischaemia in only 8 (40.0%). Radiological suspicion of bowel ischaemia was reported in a further 18 patients who had no evidence of bowel ischaemia at the time of surgery. Initial CT reports had a sensitivity and specificity for ischaemic bowel of 40.0% and 85.5%, respectively. Comparisons of clinicopathological demographics between patients with and without evidence of bowel ischaemia were performed to investigate their value in patient stratification (Table 2). Patients with ischaemic bowel were found to have a slightly higher median white cell count at diagnosis (11.4 × 10^9^/L versus 9.6 × 10^9^/L, *p* = 0.048). In contrast, no differences in arterial lactate or serum c-reactive protein levels were noted. A greater proportion of patients with bowel ischaemia had clinical signs of peritonitis, although this difference was not statistically significant (18.2% versus 8.6%, *p* = 0.238).

**Table 2:** Clinicopathological demographics in patients undergoing acute operations stratified according to the presence of ischaemic bowel

		SBO with ischaemic bowel		*p* value
		Yes	No	
Number of patients		20	124	-
Male:female		11:9	63:61	0.821
Median age in years (IQR)		77 (67–82)	73 (55–79)	0.191
Suspicion of ischaemic bowel		8 (40.0)	18 (14.5)	**0.011**
Signs of peritonitis		4 (20.0)	12 (9.7)	0.240
qSOFA ≥ 2		0 (0)	2 (1.6)	> 0.999
Median WCC (IQR)		11.4 (8.5–17.7)	9.6 (7.2–12.9)	**0.048**
Median CRP (IQR)		6.6 (3.3–50.0)	11 (4.0–45.5)	0.958
Median lactate (IQR)		1.4 (1.0–2.3)	1.2 (0.8–1.7)	0.099
AKI on admission		6 (30.0)	31 (25.0)	0.416
Number of previous operations	0	10 (50.0)	37 (29.8)	0.248
	1	5 (25.0)	41 (33.1)	
	≥ 2	5 (25.0)	46 (37.1)	
Number of previous SBO	0	18 (90.0)	108 (87.1)	0.741
	1	2 (10.0)	6 (4.8)	
	≥ 2	0 (0)	10 (8.1)	

*SBO* small bowel obstruction, *IQR* interquartile range, *qSOFA* quick sequential organ failure assessment, *WCC* white cell count, *CRP* C reactive protein, *AKI* acute kidney injury

### Closed loop obstruction

Of the 26 patients found to have closed loop obstructions at the time of surgery, radiological suspicion of a closed loop was only reported in 6 (23.1%). Radiological suspicion of a closed loop obstruction was reported in a further three patients, of whom two had simple adhesional obstructions and one had no evidence of intestinal obstruction at the time of surgery. Initial CT reports had a sensitivity and specificity for closed loop obstructions of 23.1% and 98.0%, respectively.

### Aetiology of obstruction

In terms of identifying the underlying cause of SBO, the greatest accuracy of initial CT reports was seen in patients with SBO due to hernias, with a sensitivity of 95.7% and a specificity of 98.0%. A diagnosis of adhesional SBO was confirmed in 81 patients at the time of surgery, of whom 18 had never undergone previous abdominal surgery (22.2%). Radiological suspicion of adhesional obstruction was reported in 68 of these patients (84.0%). Based on the CT report, adhesions were the suspected cause of obstruction in a further 31 patients who were found to have other aetiologies at the time of surgery, which are summarised in Table 3. When considering only those patients with adhesional SBO, the sensitivity and specificity of CT reports for identifying patients with a band adhesion were 67.5% and 47.5%, respectively.

**Table 3:** Aetiology of small bowel obstruction in patients presumed to have adhesional obstruction based on CT findings

Diagnosis	Number of patients
Closed loop obstruction	15
Malignancy	7
Small bowel stenosis	3
Venous ischaemia	2
Meckel’s diverticulum	1
Hernia (ventral)	1
Volvulus	1
No intestinal obstruction	1

## Discussion

This prospective study of initial CT reporting in patients admitted with SBO suggests that the reliability of this modality in identifying patients with small bowel obstruction with ischaemia and correctly identifying the aetiology of obstruction in everyday clinical practice is poorer than has previously been reported. The poorest performance was seen regarding the sensitivity of CT reports in identifying patients with closed loop obstructions (23.1%) and small bowel obstruction with ischaemia (40.0%), which are clear indications for acute surgical intervention.

A recent meta-analysis investigated the diagnostic accuracy of CT scanning in patients admitted with suspected SBO [10]. Including over 4000 patients from 45 separate studies, this meta-analysis reported considerable accuracy of this modality in terms of identifying bowel ischaemia (pooled sensitivity and specificity of 82% and 92%) and in identifying the underlying aetiology (pooled sensitivity for adhesions of 95%, hernias of 70%, and malignancy of 82%). However, it is worth noting that of the 45 studies included in that meta-analysis, all but 3 were retrospective studies of historical CT scans. The design of such retrospective studies, where historical images are reviewed by a small number of expert radiologists, is far removed from the clinical environment, where one is typically reliant on a single radiologist reviewing images at all hours of the day and where the decision to operate or not must be made quickly. Interestingly, discrepancies have previously been demonstrated between initial out-of-hour reports of abdominal CT scans and the final review, with bowel obstruction identified as an independent risk factor for misinterpretation [12]. The results of the current study suggest that reports of CT accuracy based on previous retrospective studies should be interpreted with caution and may not necessarily correlate with everyday clinical practice.

The use of CT scanning in patients with acute abdominal conditions is becoming more and more commonplace and is used almost ubiquitously in Denmark to diagnose SBO [4, 13]. Given the extent of its usage, strategies to improve the reliability of initial CT reporting would be of much interest. One potential strategy would be to develop a standardised format for CT reporting in patients with suspected SBO, including comments on relevant factors to aid the admitting surgeon in determining the best course of action for each individual patient. For instance, mesenteric haziness, reduced bowel enhancement, and a closed loop obstruction are predictive of bowel ischaemia, with the additional finding of free mesenteric fluid being predictive of the need for bowel resection [14]. CT features have also shown some value in predicting the success or failure of non-operative management, with the presence of fewer than two beak signs and an anterior parietal adhesion predictive of success [15]. The absence of these features could be used to identify patients who should undergo an early operation, as a failure of non-operative management is associated with significant increases in morbidity and mortality [3, 4, 16]. Additionally, the presence of a beak sign or the fat notch sign has shown value in predicting the presence of simple band adhesions, which could be used to stratify patients to laparoscopic rather than open surgery, which has benefits in terms of patient recovery time as well as short- and long-term wound-related complications [17–19]. The development and implementation of a standard reporting schema, including such factors, could reduce variability in reporting between individuals and improve the clinical applications of CT findings.

An alternative strategy to improve the reliability of CT reports could be to encourage a further imaging review by the admitting surgeon themselves. Although related to patients admitted due to trauma rather than SBO, there is some evidence to suggest that there are low levels of discrepancy between on-call surgeons and radiologists in the interpretation of abdominal CT scans [20, 21]. Given that the final responsibility in deciding whether or not to operate on a patient lies in the surgeon’s hands, routine review of acute abdominal scanning should be encouraged and clinically correlated, and surgeons should be capable of identifying factors associated with clear indications for emergency surgery, such as free intraperitoneal air or suspected bowel ischaemia. New developments in CT scanning modalities may also lead to improved accuracy for surgeons and radiologists alike. Although it was not a part of the standardised emergency care bundles used at the time of the current study, dual energy CT shows promise in the evaluation of patients with suspected bowel ischaemia [22].

It should also be noted that treatment stratification of patients with SBO should not be based on CT scanning alone, but rather that this modality should supplement the general clinical assessment. That being said, the challenges in identifying patients who have or are at risk of developing bowel ischaemia are well recognised. In the current study, the only statistically significant difference seen between patients with and without ischaemic bowel was in the white cell count at diagnosis. However, the clinical significance of this difference is questionable, given that the median value in patients with bowel ischaemia was only slightly outside of the normal range. While there has been much interest in identifying potential biomarkers for early signs of bowel ischaemia, some of which have shown promise, none of these markers are in routine clinical use [23]. The development of robust biomarkers that could further assist early patient stratification would be of major benefit.

The authors recognise the limitations of this study. The radiological assessments were based on the original written reports, but no data were collected regarding the number of individual radiologists who reported these scans. As such, no comment can be made as to the extent to which reporting accuracy varied within and between individuals. In addition, the assessment of bowel ischaemia was made based on the original operating notes and is as such a qualitative rather than quantitative assessment. A more robust design, which could be considered for future studies, would have been to perform quantitative assessments of bowel perfusion, for example using indocyanine green [24]. Finally, although the current cohort is of reasonable size when compared to previous studies [10], relatively few patients had the outcomes of interest (i.e. bowel ischaemia or closed loop obstructions). It would be of interest to see if larger prospective studies produced similar findings.

## Conclusion

The real-world accuracy of CT scanning appears to be lower than previously reported in the literature. Strategies to address this could include the development of standardised reporting schemas and to increase the surgeon’s own familiarity with relevant CT features in patients admitted with SBO.

## Data Availability

In accordance with Danish law, the data on which the findings of this study are based can not be made available for sharing.
